# Being a departmental head – what does it mean? Emphasizing the salience and shades in medical academia

**DOI:** 10.30476/jamp.2019.81476.1009

**Published:** 2020-04

**Authors:** RAJASEKHAR S.S.S.N, DINESH KUMAR. V

**Affiliations:** 1 Department of Anatomy, Jawaharlal Institute of Postgraduate Medical Education and research, Puducherry – 605006, India

**Dear Editor**

In the contemporary era, any healthcare organization needs to shape up its own management culture. This includes developing vision statement and utilizing the available resources in order to ensure the optimal level of satisfaction to its stakeholders. It has been found that the quality of healthcare services in an organization and leadership traits are directly proportional to the staff satisfaction and concurrently to patient satisfaction ( 1 ). It is a common criticism in healthcare settings that physicians are often assigned administrative roles on the sole basis of academic expertise which could not be directly equated to leadership abilities. If we consider the department as the functional unit of an institution, its function can be classified under two headings. The expertise quality which encompasses the technical aspects of its members and administrative quality denotes the non-technical organizational factors ( 1 ). The administrative quality is largely determined by the perspectives held by the head. 

Headship is an iterative process which necessitates providing a mechanism for sense-making among sub-ordinates depending upon the varying environments ( 2 ). Sense-making can be defined as the ability of the head to improve the capacity of the department by productively organizing the information/perspectives arising from various stakeholders and developing the most optimal action plan ( 3 ). The ability of sense-making gains more importance in high stake clinical departments where it is crucial to strike the right balance among teaching, research and patient service. 

Unfortunately, healthcare systems, particularly in the developing countries, fail to recognize the need for the development of leadership skills at multiple levels of the organization. The innate urge of the physicians to excel a ‘solo performer’ and function autonomously in work settings tends to make them less focused on sense making ( 4 , 5 ). In ideal sense, the ‘autonomous’ work pattern, as in a research team, creates a linear model where the decisions made in the ivory tower are passed on to the sub-ordinates ( 6 ). 

It is important to understand that neither all heads of departments nor their leadership styles are the same ( 7 ). Let us imagine three department heads, whom we have encountered in our career. Head A is a reputed hyper-optimistic clinician-scientist who has achieved plenty of accolades in his career. Head B is a pragmatic clinician with an average profile and Head C is an ideological academician who emphasizes more on the values rather than the productivity. Head A will focus only on the achievement of overarching goals with a strong belief that the outcome will always be under his control. Therefore, he tries to emotionally persuade the juniors to achieve more. Head B will have a pragmatic approach which systematically weigh the pros and cons of the situation and encourages and guides the subordinates towards achievement of their personal goals. The advantage is that he is open for arguments based on rational thinking, which is not usually seen in head A. In contrast to the two above-mentioned leadership styles, head C is custom-bound, and is usually grounded upon an archaic belief system and he shall be rejoiced by like-minded sub-ordinates. He neither persuades the followers to do more as head A nor open to rational arguments as head B. We request the readers to envisage different heads they have faced in their career and introspect upon the various traits they have been possessing. 

Conventionally, the organizational nature of the environment and the concept of order determine the functionality of headship ( 8 ). The organizational nature of the department depends on factors such as vision model, level of conflicts, interdependence of members, and political climate. For example, head A shall function better in a resourceful and a junior heavy department because the members can be easily persuaded by the head’s charisma, as a result of which devising of attainable future goals prompting a collective action becomes easier. On other hand, head B would be ideal for the departments where members tend to be proactive, autonomous and ambitious since the role of head is to orchestrate the smooth running of the department by reducing the conflicts and analyzing the ripples caused by each decision at multiple levels. However, the headship skills which usually produce the desired results in a structurally organised hierarchical system will not produce results in a dysfunctional hierarchy and rebellious situation, which results in a chaos. A dysfunctional system will precipitate in interpersonal conflict and lack of trust among the members. In such situations, a head of department should try to build a comprehensive organisational structure that facilitates the alignment of the individual goals with that of the institutional goals. 

In conclusion, we could state that there is a crucial role of leadership training program in sculpting an effective departmental head out of a physician ( 9 ). This can be achieved by experiential learning processes, such as case-based discussions, strategic mentorship and role modelling, which could broaden the mind-set and increase the organizational commitment of individuals chairing the department. Upon assuming the chair, the professoriate could no longer be self-centred upon his/her own endeavours, should not pressurize the juniors with their past beliefs or own ideas and never place the authorized power over purpose. The strength of headship doesn’t depend upon the ‘silence’ created among the members by virtue of dysfunctional hierarchy. Rather, it is bestowed in bringing out the best of all members and aiding in carrying the legacy forward. We believe that this piece would add to the small pool of studies available in the field of physician leadership and training.
